# RETRACTION: miR‐25‐3p Reverses Epithelial‐Mesenchymal Transition via Targeting Sema4C in Cisplatin‐Resistance Cervical Cancer Cells

**DOI:** 10.1111/cas.70083

**Published:** 2025-04-30

## Abstract

**RETRACTION**: J. Song and Y. Li, “miR‐25‐3p Reverses Epithelial‐Mesenchymal Transition via Targeting Sema4C in Cisplatin‐Resistance Cervical Cancer Cells,” *Cancer Science* 108, no. 1 (2017): 23–31, https://doi.org/10.1111/cas.13104.

The above article, published online on 01 December 2016 in Wiley Online Library (wileyonlinelibrary.com), has been retracted by agreement between the journal Editor‐in‐Chief, Masanori Hatakeyama; the Japanese Cancer Association; and John Wiley & Sons Australia Ltd. The retraction has been agreed following an investigation into concerns raised by a third party, which revealed inappropriate duplication of image panels (Figures 1b, 2a, b and 3c) between this and several other articles published previously or in the same year by a different group of authors, in a different scientific context. Given the extent of the identified issues, the editors have lost confidence in the data presented and the article's conclusions can no longer be considered reliable. The authors and their institute have been informed of the concerns and the decision to retract but they remained unresponsive.

**RETRACTION**: J. Song and Y. Li, “miR‐25‐3p Reverses Epithelial‐Mesenchymal Transition via Targeting Sema4C in Cisplatin‐Resistance Cervical Cancer Cells,” *Cancer Science* 108, no. 1 (2017): 23–31, https://doi.org/10.1111/cas.13104.

The above article, published online on 01 December 2016 in Wiley Online Library (wileyonlinelibrary.com), has been retracted by agreement between the journal Editor‐in‐Chief, Masanori Hatakeyama; the Japanese Cancer Association; and John Wiley & Sons Australia Ltd. The retraction has been agreed following an investigation into concerns raised by a third party, which revealed inappropriate duplication of image panels (Figures 1b, 2a, b and 3c) between this and several other articles published previously or in the same year by a different group of authors, in a different scientific context. Given the extent of the identified issues, the editors have lost confidence in the data presented and the article's conclusions can no longer be considered reliable. The authors and their institute have been informed of the concerns and the decision to retract but they remained unresponsive.

